# Effects of growth hormone/estrogen/androgen on COVID-19-type proinflammatory responses in normal human lung epithelial BEAS-2B cells

**DOI:** 10.1186/s12860-022-00442-5

**Published:** 2022-09-29

**Authors:** Zemin Zhu, Zhijian Zhao, Xun Chen, Zhou Chu, Yi He, Yingzheng Tan, Juan Zhou, Caixi Tang

**Affiliations:** 1 Department of Hepatobiliary and Pancreatic Surgery, Zhuzhou Central Hospital,, Zhuzhou, China; 2 Department of Cardiovascular Medicine, Zhuzhou Central Hospital, Zhuzhou, China; 3 Department of Infectious Diseases, Zhuzhou Central Hospital, Zhuzhou, China

**Keywords:** Growth hormone, Sex hormones, COVID-19, Proinflammatory responses, BEAS-2B cells

## Abstract

**Background:**

COVID-19 is a disease caused by SARS-CoV-2, which can cause mild to serious infections in humans. We aimed to explore the effect of growth hormone (GH)/estrogen/androgen in normal human lung epithelial BEAS-2B cells on COVID-19-type proinflammatory responses.

**Methods:**

A BEAS-2B COVID-19-like proinflammatory cell model was constructed. After that, the cells were treated with GH, 17β-estradiol (E2), and testosterone (Tes) for 24 h. CCK-8 assays were utilized to evaluate cell viability. The mRNA expression of *ACE2*, *AGTR1*, *TMRRSS2*, and *ISG15* and the protein expression of ACE2, AGTR1, TMRRSS2, and ISG15 were measured by qRT‒PCR and Western blotting, respectively. ELISAs were performed to determine IL-6, MCP-1, MDA and SOD expression. Flow cytometry was used to measure ROS levels. Finally, MAPK/NF-κB pathway-related factor expression was evaluated.

**Results:**

The COVID-19-type proinflammatory model was successfully constructed, and 1000 ng/mL RBD treatment for 24 h was selected as the condition for the model group for subsequent experiments. After RBD treatment, cell viability decreased, the mRNA expression of *ACE2*, *AGTR1*, *TMRRSS2*, and *ISG15* and the protein expression of ACE2, AGTR1, TMRRSS2, and ISG15 increased, IL-6, MCP-1, MDA and ROS levels increased, and MDA levels decreased. The mRNA levels of *MAPK14* and *RELA* increased, but the protein levels did not change significantly. In addition, phospho-MAPK14 and phospho-RELA protein levels were also increased. Among the tested molecules, E2 had the most pronounced effect, followed by GH, while Tes showed the opposite effect.

**Conclusion:**

GH/E2 alleviated inflammation in a COVID-19-type proinflammatory model, but Tes showed the opposite effect.

## Introduction

The coronavirus disease 2019 (COVID-19) pandemic, caused by severe acute respiratory syndrome coronavirus 2 (SARS-CoV-2), poses a serious public health threat globally [1]. SARS-CoV-2 usually spreads through respiratory droplets. The average incubation period is 6.4 days; most patients have mild symptoms, and a few patients develop severe hypoxia, requiring hospitalization and mechanical ventilation [2]. Age, sex, obesity and smoking, and alterations in disease state-related receptor expression may correlate with the incidence and severity of COVID-19 [3]. Clinical data suggest that SARS-CoV-2 infection is generally more severe in adults than in children [4]. It has been speculated that the lower clinical severity in children may be influenced by differential expression of SARS-CoV-2’s major host functional receptor, angiotensin-converting enzyme 2 (ACE2), but the data remain conflicting [4]. SARS-CoV-2 enters host cells via ACE2, which is highly expressed in the heart, kidney and lung and shed into plasma [5]. Compared with women, men have a higher incidence, severity, and mortality from COVID-19 [6], but the underlying factors contributing to this sex difference are still being studied.

Differences in the levels of growth hormone (GH) and sex hormones are known to be one of the most significant differences between children and adults. Estrogen and androgen may be closely related to the expression of ACE2 [7]. Emerging evidence suggests that the virus also targets male and female reproductive organs that express ACE2, its main receptor [8]. Reductions in sex steroids (especially in women) and GH (both of which play key roles in immune regulation) are key contributors to the severity of SARS-CoV-2 in older adults [9]. Insulin-like growth factor-1 (IGF1) affects the immune system by controlling the endocrine system [10]. There may be an association between low IGF1 (and possibly GH) and poor prognosis in COVID-19 patients [11]. There is clear evidence that dysregulation of the immune system is a key factor in determining SARS-CoV-2 severity and its association with adult-onset GH deficiency [12]. Galganska H et al. reported that the receptor binding domain of the SARS-CoV-2 spike protein stimulates a COVID-19-like proinflammatory response [13]. In addition, testosterone (Tes), GH, and estrogen are all associated with the TLR4 inflammatory pathway [14–16]. However, the effect of GH/estrogen/androgen on COVID-19-type proinflammatory responses is unknown.

Therefore, based on the above background, we wanted to study the effects of GH/estrogen/androgen in BEAS-2B cells on COVID-19-type proinflammatory responses. Our research may provide novel ideas and references for preventing and treating COVID-19.

## Materials and methods

### Cell treatment

Normal human lung epithelial BEAS-2B cells were provided by BLUEFBIO (BFN608009328, China) and cultured in DMEM high glucose medium with 10% FBS and 1% double antibiotics. A COVID-19-type proinflammatory cell model was constructed [13, 17, 18], and the cells were grouped into the control group (normal cultured cells + solvent control for 12 and 24 h), RBD-500 group (BEAS-2B cells treated with 500 ng/mL recombinant SARS-CoV-2 spike RBD protein with His tag (RBD) for 12 and 24 h), and RBD-1000 group (BEAS-2B cells treated with 1000 ng/mL RBD for 12 and 24 h). To study the effects of GH/estrogen/androgen on COVID-19-type proinflammatory responses in BEAS-2B cells, the cells were further divided into the control group (normal cultured cells + solvent control treatment for 24 h), RBD group (cells treated with 1000 ng/mL RBD for 24 h), RBD + GH group (cells treated with 1000 ng/mL RBD and 200 ng/mL GH simultaneously for 24 h), RBD + E2 group (cells treated with 1000 ng/mL RBD and 1 nM 17β-estradiol (E2) simultaneously 24 h [6]), and RBD + Tes group (cells treated with 1000 ng/mL RBD and 10 nM Tes simultaneously treated for 24 h [6]).

### Cell counting Kit-8 (CCK-8) assay

Different groups of BEAS-2B cells were digested, counted and seeded in a 96-well plate at a density of 1 × 10^4^ cells/well, with 100 µL per well. The cell count assay was performed in replicates. Each group had 3 replicate wells. After culturing adherent cells, 10 µL/well of CCK-8 (#NU679, DOJINDO, Japan) was added, and CCK-8 solution was prepared with complete medium. After incubation at 37 °C and 5% CO_2_, the absorbance value at 450 nm was analyzed on a Bio-Tek microplate reader.

### Quantitative real-time PCR (qRT‒PCR)

Total RNA was extracted by TRIzol (#15596026, Thermo, USA) and reverse transcribed into cDNA with a cDNA reverse transcription kit (#CW2569, Beijing ComWin Biotech, China). Subsequently, real-time PCR was performed on a fluorescence quantitative PCR instrument (QuantStudio1, Thermo, USA) using Ultra SYBR Mixture (#CW2601, Beijing ComWin Biotech, China). The reaction system was 30 µL, including 2 µL reverse transcription product cDNA, 1 µL Primer R (10 µM), 1 µL Primer F (10 µM), 11 µL ddH_2_O, and 15 µL 2× SYBR Green PCR Master Mix. The reaction conditions were as follows: predenaturation at 95°C for 10 min, and 40 cycles of denaturation at 95°C for 15 s and annealing at 60°C for 30 s. The experiment was repeated three times. Using ACTB as the internal reference gene, the gene levels were calculated by the 2^−ΔΔCt^ method, where △△Ct=△Ct experimental group-△Ct control group, and △Ct = Ct (target gene)-Ct (internal reference). The primer sequences were as follows: *ACE2*-F: 5’-GGACACTGAGCTCGCTTCTG-3’, *ACE2*-R: 5’-CCTTTGAACTTGGGTTGGGC-3’; *AGTR1*-F: 5’-CGGGGCGCGGGTTTG-3’, *AGTR1*-R: 5’-TACACCTGGTGCCGACTTTC-3’; *TMPRSS2*-F: 5’-ATACAAGCTGGGGTTCTGGC-3’, *TMPRSS2*-R: 5’-TAGCCGTCTGCCCTCATTTG-3’; *ISG15*-F: 5’-GGTGGACAAATGCGACGAAC-3’, *ISG15*-R: 5’-TCGAAGGTCAGCCAGAACAG-3’; *MAPK14*-F: 5’-ACAGGATGCCAAGCCATGAG-3’, *MAPK14*-R: 5’-GATCGGCCACTGGTTCATCA-3’; *RELA* -F: 5’-CGCCTGTCCTTTCTCATCCCAT-3’, *RELA*-R: 5’-CCTCTTTCTGCACCTTGTCACAC-3’; *ACTB*-F: 5’-ACCCTGAAGTACCCCATCGAG-3’, *ACTB*-R: 5’-AGCACAGCCTGGATAGCAAC-3’.

### Western blotting

Total protein was extracted from 5.3 × 10^6^ BEAS-2B cells using a RIPA kit (P0013B, Beyotime, China). After the protein concentration was determined, proteins were separated by 10% SDS‒PAGE electrophoresis. Proteins were transferred to PVDF membranes by electrotransfer. For primary antibodies, we used anti-ACE2 (21115-1-AP, 1:1000, Proteintech, USA), anit-AGTR1 (25343-1-AP, 1:500, Proteintech, USA), anti-TMRRSS2 (ab92323, 1:1000, Abcam, UK), anti-ISG15 (15981-1-AP, 1:500, Proteintech, USA), anti-phospho-MAPK14 (28796-1-AP, 1:1000, Proteintech, USA), anti-MAPK14 (14064-1-AP, 1:1000, Proteintech, USA), anti-phospho-RELA (ab76302, 1:1000, Abcam, UK), anti-RELA (10745-1-AP, 1:1000, Proteintech, USA), and anti-ACTB (66009-1-Ig, 1:5000, Proteintech, USA) antibodies. The membranes were then incubated with HRP-conjugated secondary antibodies. β-actin was used as the internal reference. The membrane was immersed in ECL chemiluminescent solution (K-12,045-D50, Advantesta, USA) for luminescence visualization. Protein bands were detected using a chemiluminescence imaging system (ChemiScope 6100, Clinx, China). The experiment was repeated three times.

### Enzyme-linked immunosorbent assay (ELISA)

IL-6 (KE00007, Proteintech, USA), MCP-1 (KE20009, Proteintech, USA), MDA (#A003-1, Nanjing Jiancheng Bioengineering Institute, China), and SOD (#A001-3, Nanjing Jiancheng Bioengineering Institute, China) quantitative ELISA kits were used to detect related factor levels according to the manufacturer’s recommendations. A DYNATECHM 7000 microplate reader was used to calculate each indicator concentration by forming a standard curve through the constant value. The experiment was repeated three times.

### Flow cytometry (FCM)

The level of ROS was assessed according to the ROS assay kit (S0033S, Beyotime, China). First, DCFH-DA (stock concentration 10 mM) was diluted 1:1000 in serum-free medium to a final concentration of 10 µM. Then, the cells treated as indicated above were removed from the cell culture medium, and DCFH-DA was added to cover the cells, followed by incubation in a 37 °C cell incubator for 20 min. The samples were collected by trypsinization. Finally, FCM (A00-1-1102, Beckman, USA) detection was performed using an excitation wavelength of 488 nm and an emission wavelength of 525 nm. The level of ROS was analyzed with CyExpert software. The experiment was repeated three times.

### Statistical analysis

GraphPad Prism 8.0 software was applied for statistical analysis. The experiments were repeated three times. Measurement data are expressed as the mean ± standard deviation. The experiments were repeated three times. The significance of three or more groups was compared by one-way ANOVA. Tukey’s test was adopted for postmortem testing. P < 0.05 indicated a statistically significant difference.

## Results

### Construction of a COVID-19-type proinflammatory model

**Fig. 1:**
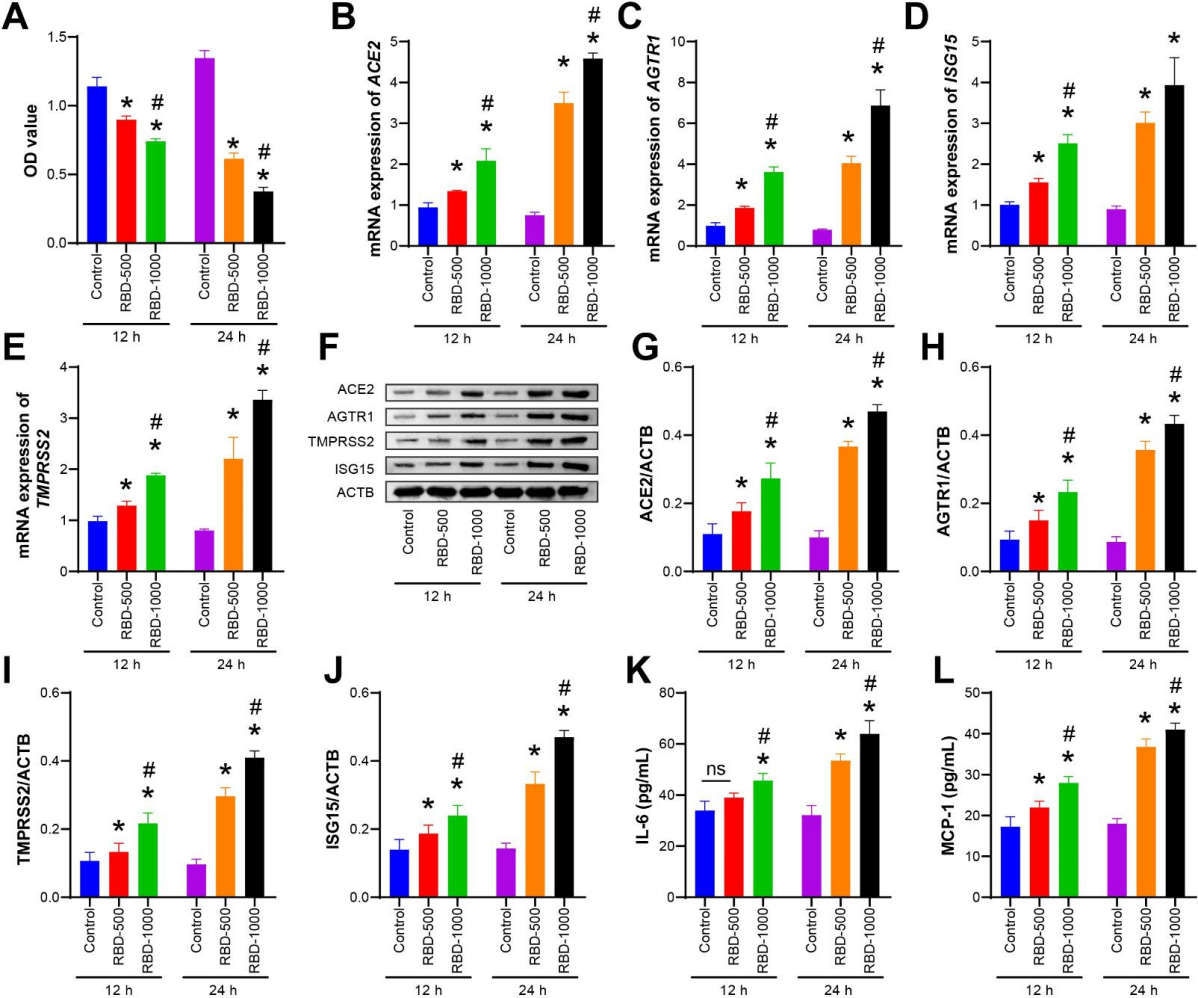
**Construction of a COVID-19-type proinflammatory model. A**. Cell viability was assessed by a CCK-8 assay at 12 and 24 h. **B-E**. qRT‒PCR detection of the mRNA expression of *ACE2*, *AGTR1*, *TMRRSS2*, and *ISG15* at 12 and 24 h. **F-J**. Western blot detection of the protein expression of ACE2, AGTR1, TMRRSS2, and ISG15 at 12 and 24 h. **K-L**. ELISAs were used to measure IL-6 and MCP-1 levels. The experiments were repeated three times. The significance of three or more groups was compared by one-way ANOVA. Tukey’s test was adopted for postmortem testing. * P < 0.05 vs. control, # P < 0.05 vs. RBD-500.

First, a COVID-19-type proinflammatory model was established, and cell viability was evaluated by CCK-8 assay. The cell viability of the RBD-500 and RBD-1000 groups decreased at 12 and 24 h compared with that of the control group (Fig. 1 A). Figure 1B J showed that the mRNA expression of *ACE2*, *AGTR1*, *TMRRSS2*, and *ISG15* and the protein expression of ACE2, AGTR1, TMRRSS2, and ISG15 both increased at 12 and 24 h in the RBD-500 group and the RBD-1000 group. Among these, the effect of 1000 ng/mL RBD treatment for 24 h was more significant. Finally, the levels of MCP-1 and IL-6 were assessed by ELISA. After RBD treatment, both MCP-1 and IL-6 levels were increased, and the increase was more evident after 1000 ng/mL RBD treatment for 24 h (Fig. 1 K-1 L). Therefore, 1000 ng/mL RBD treatment for 24 h was selected as the condition for the model group for follow-up experiments.

### The role of GH/E2/Tes in a COVID-19-type proinflammatory model

**Fig. 2:**
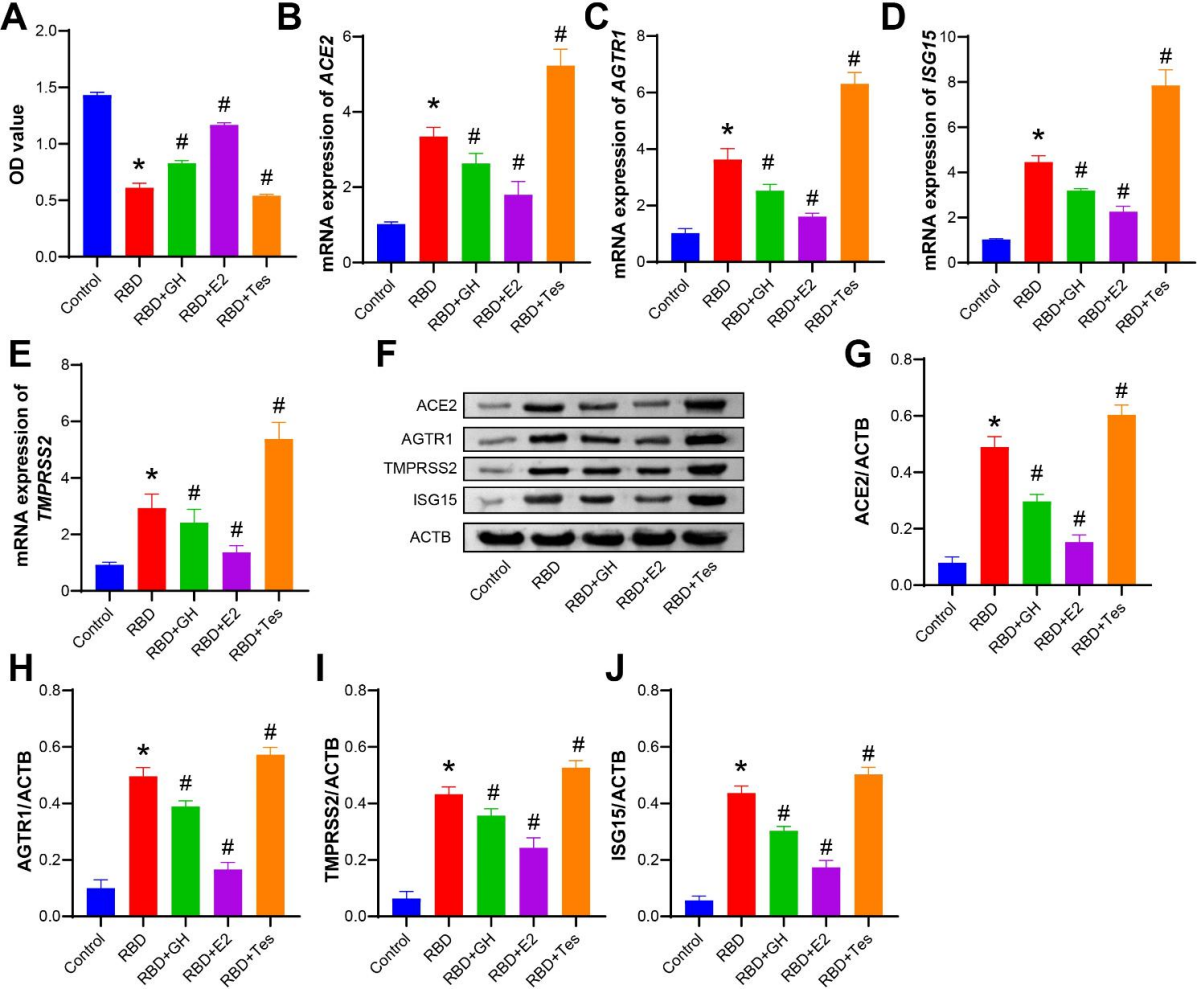
**The role of GH/E2/Tes in a COVID-19-type proinflammatory model** BEAS-2B cells were treated with 1000 ng/mL RBD and 200 ng/mL GH, 1 nM E2, or 10 nM Tes simultaneously for 24 h. **A**. CCK-8 assay for cell viability. **B-E**. qRT‒PCR detection of the mRNA expression of *ACE2*, *AGTR1*, *TMRRSS2*, and *ISG15*. **F-J**. Western blot detection of ACE2, AGTR1, TMRRSS2, and ISG15 protein expression. The experiments were repeated three times. The significance of three or more groups was compared by one-way ANOVA. Tukey’s test was adopted for postmortem testing. * P < 0.05 vs. control, # P < 0.05 vs. RBD, ns: no significance

To study the effect of GH/estrogen/androgen in BEAS-2B cells on COVID-19-type proinflammatory responses, we incubated the cells with GH, E2, and Tes. The CCK-8 assay showed that the cell viability decreased after RBD treatment, and the cell viability recovered after adding GH and E2. Among these, E2 had the best effect, followed by GH. While Tes had the opposite effect (Fig. 2 A). Figure 2B -2J showed that the mRNA expression of *ACE2*, *AGTR1*, *TMRRSS2*, and *ISG15* and the protein expression of ACE2, AGTR1, TMRRSS2, and ISG15 were all increased after RBD treatment. After adding GH and E2, the mRNA expression of *ACE2*, *AGTR1*, *TMRRSS2*, and *ISG15* and the protein expression of ACE2, AGTR1, TMRRSS2, and ISG15 both decreased. Among these, E2 demonstrated the best effect, followed by GH. But Tes had the opposite effect.

### GH/E2 alleviated inflammation in a COVID-19-type proinflammatory model

**Fig. 3:**
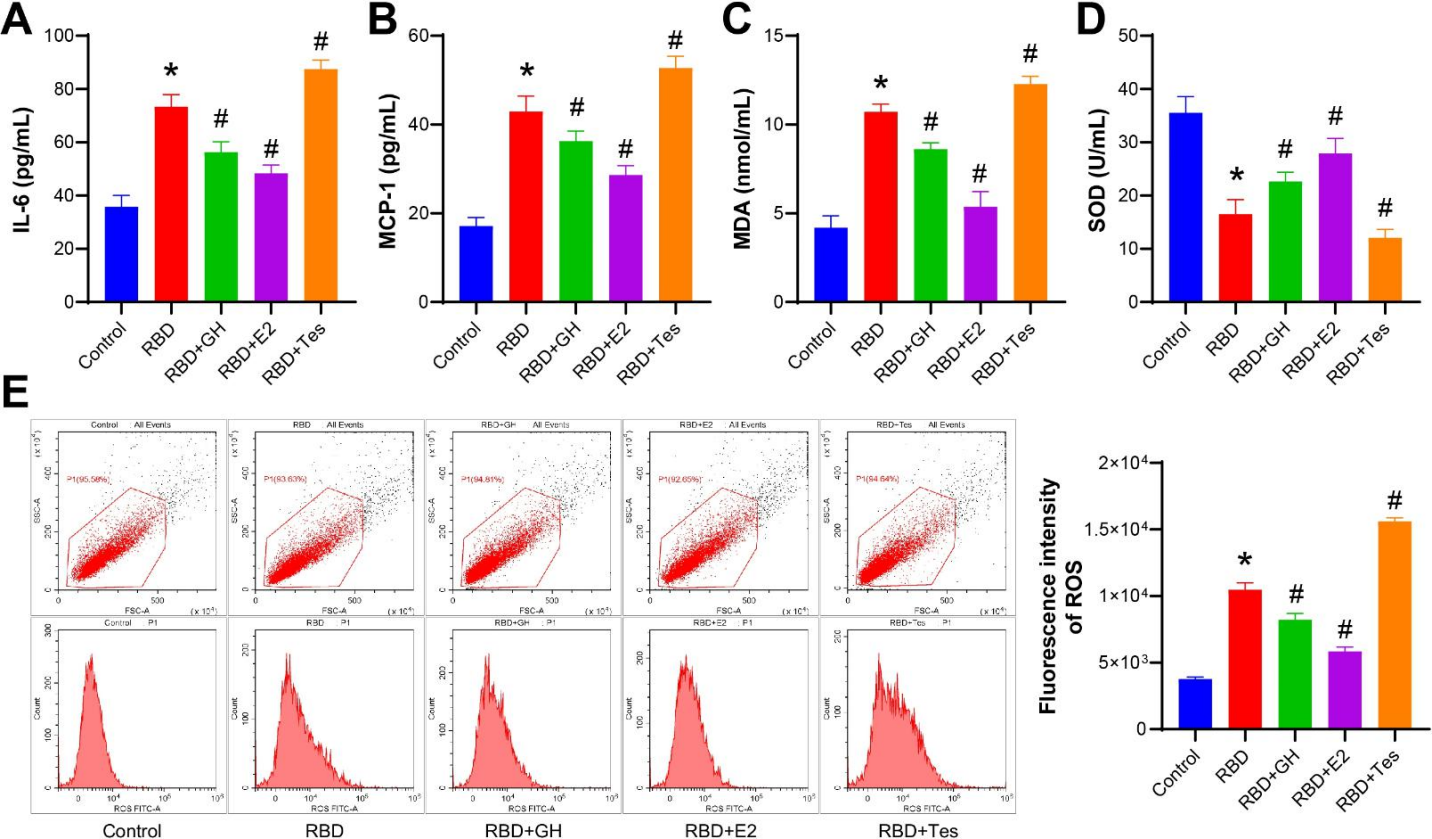
**GH/E2 alleviated inflammation in a COVID-19-type proinflammatory model.** BEAS-2B cells were treated with 1000 ng/mL RBD and 200 ng/mL GH, 1 nM E2, or 10 nM Tes simultaneously for 24 h. **A-B**. ELISAs were performed to measure IL-6 and MCP-1 levels. **C-D**. MDA and SOD levels were evaluated by ELISA. **E**. ROS levels were measured by FCM. The experiments were repeated three times. The significance of three or more groups was compared by one-way ANOVA. Tukey’s test was adopted for postmortem testing. * P < 0.05 vs. control, # P < 0.05 vs. RBD.

Next, we examined markers related to inflammation and oxidative stress. As shown in Fig. 3 A-3E, after RBD treatment, IL-6, MCP-1, MDA and ROS levels were all increased, and the level of SOD was decreased. After adding GH and E2, IL-6, MCP-1, MDA and ROS levels all decreased, and the level of SOD increased. Among these, E2 had the most obvious therapeutic effect, followed by GH. However, Tes had the opposite effect.

### GH/E2 could regulate the MAPK/NF-κB pathway

**Fig. 4:**
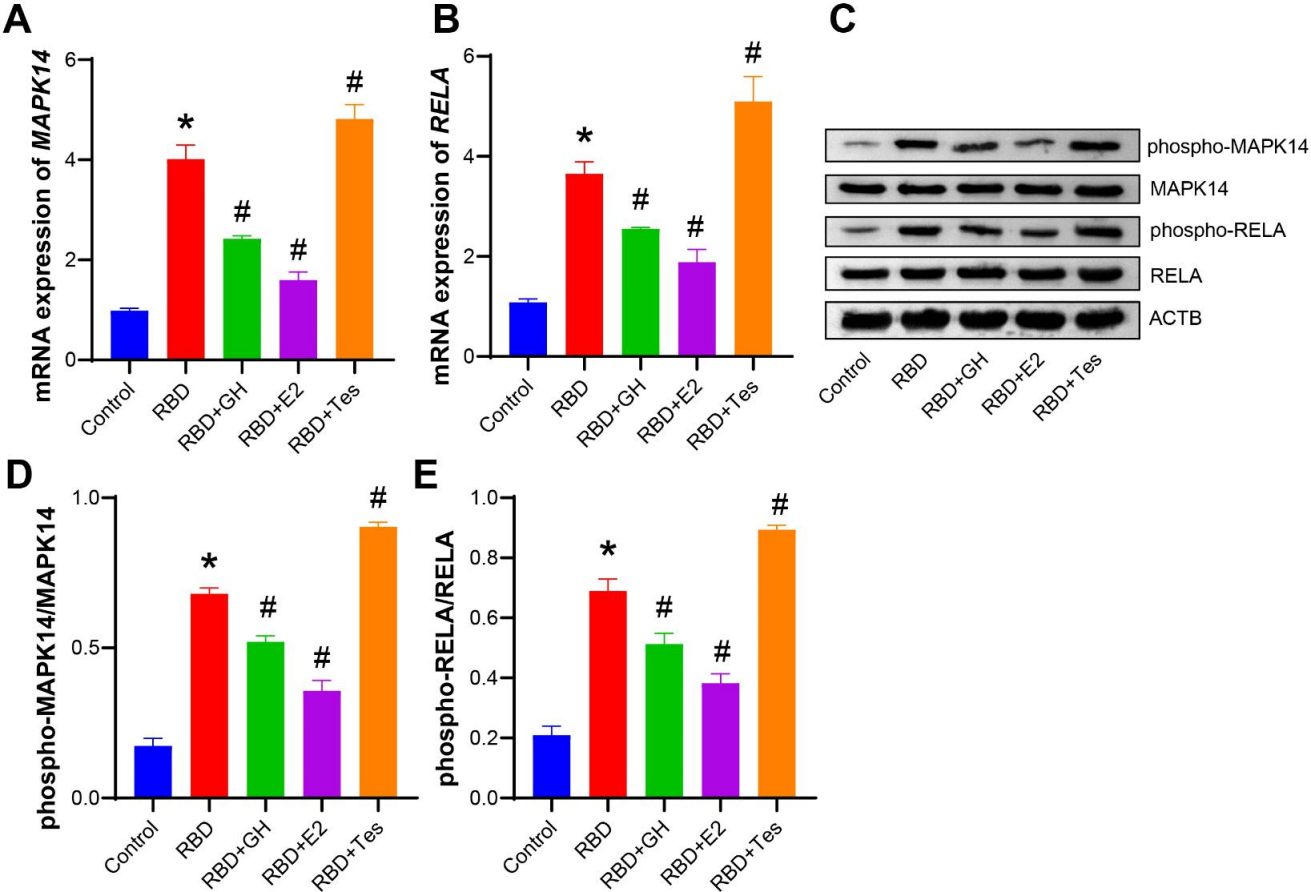
**GH/E2 could regulate the MAPK/NF-κB pathway.** BEAS-2B cells were treated with 1000 ng/mL RBD and 200 ng/mL GH, 1 nM E2, or 10 nM Tes simultaneously for 24 h. **A-B**. The mRNA expression of *MAPK14* and *RELA* in the MAPK/NF-κB pathway was measured by qRT‒PCR. **C-E**. The protein expression of phospho-MAPK14, MAPK14, phospho-RELA and RELA in the MAPK/NF-κB pathway was evaluated by Western blotting. The experiments were repeated three times. The significance of three or more groups was compared by one-way ANOVA. Tukey’s test was adopted for postmortem testing. * P < 0.05 vs. control, # P < 0.05 vs. RBD.

Finally, we examined levels of MAPK/NF-κB pathway-related factors. After RBD treatment, the mRNA expression of *MAPK14* and *RELA* increased, while the protein expression did not change significantly. Simultaneously, the protein expression of phospho-MAPK14 and phospho-RELA was also increased. After the addition of GH and E2, the mRNA expression of *MAPK14* and *RELA* decreased, while the protein expression did not change significantly. In addition, the protein expression of phospho-MAPK14 and phospho-RELA was also decreased (Fig. 4 A-4E). Among these, the role of E2 was the most obvious, followed by GH. But Tes had the opposite effect.

## Discussion

COVID-19 is a novel infectious disease characterized by atypical pneumonia caused by SARS-CoV-2, which can cause mild to severe infection in humans [19, 20]. COVID-19 has now spread rapidly around the world, posing severe challenges to health care facilities and medical infrastructure. In this research, we studied the effects of GH/estrogen/androgen in normal human lung epithelial BEAS-2B cells on COVID-19-type proinflammatory responses. We found that after E2/GH treatment, cell viability was increased, the mRNA expression of *ACE2*, *AGTR1*, *TMRRSS2*, and *ISG15* and the protein expression of ACE2, AGTR1, TMRRSS2, and ISG15 were decreased, and inflammation was alleviated. In addition, E2/GH could regulate the MAPK/NF-κB pathway.

ACE2 is the receptor of the SARS-CoV-2 spike protein [9]. SARS-CoV-2 binds to ACE2 on host cells via its surface spike protein [21], triggering complex molecular events that lead to excessive inflammation [22]. COVID-19 also targets male and female reproductive organs that express its primary receptor, ACE2, although it is unclear whether this has any effect on human fertility [8]. AGTR1 is a receptor required to support the angiotensin signaling cascade, and TMPRSS2 is a protease capable of promoting viral fusion [23]. During COVID-19, TMPRSS2 is widely expressed in respiratory and gastrointestinal tissues [24]. Moreover, ISG15-dependent activation of the sensor MDA5 was shown to be antagonized by SARS-CoV-2 papain-like protease to evade host innate host immunity [25]. In this study, we found that after E2/GH treated, the mRNA expression of *ACE2*, *AGTR1*, *TMRRSS2*, and *ISG15* and the protein expression of ACE2, AGTR1, TMRRSS2, and ISG15 decreased. This suggested that E2/GH may have a therapeutic effect on COVID-19.

GH deficiency is a common factor in all vulnerable COVID-19 patient populations [26]. Dhindsa S et al. found that lower Tes concentrations during hospitalization were related to elevated disease severity and inflammation in men in a single-center cohort study of COVID-19 patients [27]. Sex hormones reduce the inflammatory response and regulate the expression of ACE2 [28]. The androgen receptor is the transcriptional promoter of TMPRSS2, which can promote SARS-CoV-2 entry into the host [29]. Tes may make men more susceptible to widespread COVID-19 infection than estrogen. Low serum testosterone levels, which should be considered a feature of the hormonal milieu in severe patients, may predispose men, especially older men, to a poor prognosis or death [30]. In contrast, E2 treatment results in serum concentrations equivalent to ovulation or pregnancy, with beneficial immunomodulatory and anti-inflammatory effects in mice and humans [30, 31]. In the present study, after treatment of BEAS-2B cells with E2/GH, the IL-6, MCP-1, MDA and ROS levels were all decreased, and SOD was increased, suggesting that E2/GH treatment alleviated COVID-19-type proinflammatory responses.

IL-6 is a proinflammatory factor released in severely ill COVID-19 patients. This cytokine activates the MAPK/NF-κB-IL-6 pathway [31]. Ma Q et al. reported that Liu Shen capsules can inhibit SARS-CoV-2 infection by downregulating the expression of inflammatory cytokines induced by the virus in vitro, regulating the activity of the MAPK/NF-κB signaling pathway, and inhibiting SARS-CoV-2 infection and are promising new coronavirus control drug candidates [32]. In addition, Sharma VK et al. reported that nanocurcumin effectively inhibited SARS-CoV-2 spike protein-induced cytokine storm by inhibiting the MAPK/NF-κB pathway in epithelial cells [33]. In this study, we found that E2/GH regulated the MAPK/NF-κB pathway. Among these, the role of E2 was the most obvious, followed by GH. But Tes had the opposite effect.

However, this study had some limitations. We only performed in vitro cell experiments to verify the role of GH/estrogen/androgen in a COVID-19-like proinflammatory model. In vivo animal and human experiments are still lacking. Due to the limitations of time and funding, we cannot solve this problem at present. In the future, we will further explore the role of GH/estrogen/androgen in the COVID-19-like proinflammatory model in animals and humans.

In conclusion, our results showed that E2/GH treatment of BEAS-2B cells increased cell viability, decreased the mRNA expression of *ACE2*, *AGTR1*, *TMRRSS2*, and *ISG15* and the protein expression of ACE2, AGTR1, TMRRSS2, and ISG15, and alleviated inflammation. In addition, E2/GH could regulate the MAPK/NF-κB pathway. Our research provides a reference and basis for the diagnosis of COVID-19 and a new strategy for treating COVID-19.

## Data Availability

All data included in this study are available upon request by contact with the first author or corresponding author.
